# Lung Microbiome in Idiopathic Pulmonary Fibrosis and Other Interstitial Lung Diseases

**DOI:** 10.3390/ijms23020977

**Published:** 2022-01-17

**Authors:** Francesco Amati, Anna Stainer, Marco Mantero, Andrea Gramegna, Edoardo Simonetta, Giulia Suigo, Antonio Voza, Anoop M. Nambiar, Umberto Cariboni, Justin Oldham, Philip L. Molyneaux, Paolo Spagnolo, Francesco Blasi, Stefano Aliberti

**Affiliations:** 1 Department of Biomedical Sciences, Humanitas University, Via Rita Levi Montalcini 4, 20072 Pieve Emanuele, Italy; anna.stainer@hunimed.eu (A.S.); Giulia.suigo@humanitas.it (G.S.); antonio.voza@humanitas.it (A.V.); stefano.aliberti@unimi.it (S.A.); 2 Respiratory Unit, IRCCS Humanitas Research Hospital, Via Manzoni 56, 20089 Rozzano, Italy; 3 Department of Pathophysiology and Transplantation, University of Milan, 20122 Milan, Italy; marco.mantero@unimi.it (M.M.); andrea.gramegna@unimi.it (A.G.); edoardo.simonetta@unimi.it (E.S.); francesco.blasi@unimi.it (F.B.); 4 Internal Medicine Department, Respiratory Unit and Cystic Fibrosis Adult Center, Fondazione IRCCS Ca’ Granda Ospedale Maggiore Policlinico, 20122 Milan, Italy; 5 Emergency Medicine Unit, IRCCS Humanitas Research Hospital, Via Manzoni 56, 20089 Rozzano, Italy; 6 Division of Pulmonary and Critical Care, Department of Medicine, University of Texas Health San Antonio, South Texas Health Care System, San Antonio, TX 78229, USA; nambiar@uthscsa.edu; 7 Department of General and Thoracic Surgery, Humanitas Research Hospital, 20089 Rozzano, Italy; umberto.cariboni@humanitas.it; 8 Division of Pulmonary, Critical Care and Sleep Medicine, University of California Davis, Sacramento, CA 95616, USA; joldham@ucdavis.edu; 9 National Heart and Lung Institute, Imperial College London, London SW7 2AZ, UK; p.molyneaux@imperial.ac.uk; 10 Respiratory Disease Unit, Department of Cardiac, Thoracic, Vascular Sciences and Public Health, University of Padova, 35128 Padova, Italy; paolo.spagnolo@unipd.it

**Keywords:** microbiome, interstitial lung diseases, treatable traits

## Abstract

Interstitial lung diseases represent a heterogeneous and wide group of diseases in which factors leading to disease initiation and progression are not fully understood. Recent evidence suggests that the lung microbiome might influence the pathogenesis and progression of interstitial lung diseases. In recent years, the utilization of culture-independent methodologies has allowed the identification of complex and dynamic communities of microbes, in patients with interstitial lung diseases. However, the potential mechanisms by which these changes may drive disease pathogenesis and progression are largely unknown. The aim of this review is to discuss the role of the altered lung microbiome in several interstitial lung diseases. Untangling the host–microbiome interaction in the lung and airway of interstitial lung disease patients is a research priority. Thus, lung dysbiosis is a potentially treatable trait across several interstitial lung diseases, and its proper characterization and treatment might be crucial to change the natural history of these diseases and improve outcomes.

## 1. Introduction

Interstitial lung diseases (ILDs) represent a heterogeneous group of diseases encompassing more than 200 entities that affect the lung parenchyma with inflammation and/or fibrosis [1]. Factors leading to disease initiation and progression are not fully understood for most of ILDs, although the interaction between genetic and environmental factors is believed to be a major driver of disease pathogenesis [2]. While a number of genetic variants that are potentially pathogenic are well recognized, the environmental triggers remain largely unidentified [3,4,5].

Until recently, the lung has been considered “sterile” because traditional, culture-based techniques are not sufficiently sensitive to isolate and identify a large number of microbes [6]. Culture-independent methodologies, such as DNA sequencing, have allowed the identification of complex and dynamic communities of microbes, which coexist in the lung of healthy subjects and those with chronic respiratory diseases [7]. The respiratory tract of healthy individuals harbors a natural community of microbes, and mounting evidence links alteration in these communities to the pathogenesis and progression of several airway diseases, such as asthma, bronchiectasis, and chronic obstructive pulmonary disease (COPD) [8,9,10]. A growing body of evidence also points toward the role of dysbiosis of the lung microbiome as a contributor to the development and progression of ILDs and, in particular, idiopathic pulmonary fibrosis (IPF) [11]. The aim of this review is to discuss the role of the lung microbiome in the pathogenesis and progression of ILDs with emphasis on potential interventions.

## 2. Healthy Lung Microbiome

Over the last two decades, the microbiome has gained popularity in the scientific community. Joshua Lederberg was among the first to introduce the term “microbiome” to highlight the importance of “the ecological community of commensal, symbiotic, and pathogenic microorganisms that share our body space and have been all but ignored as determinants of health and disease” [12]. It soon became evident that the microbiome was a fascinating area that integrates a new perspective on the coexistence of microorganisms in the human body into mechanisms that, if deranged, may lead to disease development. Phenotyping and endotyping patients based on microbiome analysis might have prognostic and theragnostic benefits [13]. However, historically, the lungs were considered sterile based on the results of culture-based studies and excluded from the five major body sites (gastrointestinal tract, mouth, vagina, skin, and nose) in the original Human Microbiome Project [14]. Culture-independent techniques changed the landscape of the lung microbiome scenario. Molecular-based techniques, such as 16S ribosomal (r) RNA and metagenomic sequencing, showed that lungs contain a large number of communities of microbes, even without clinical evidence of infection [15,16,17]. The rapid development of this field required clinical researchers to adopt a new language to describe lung microbial communities and their impact on lung health. Updated microbiome terminology is detailed in Table 1 [18]. From birth onwards, the lungs are repeatedly exposed to diverse microorganisms. This diversity of bacterial exposure, the interaction with the environment, and any treatments administered may play a fundamental role in determining susceptibility to pulmonary disease [17,19]. In a healthy individual, a load of microorganisms in the lungs is equivalent to 10^3^–10^5^ bacteria per gram of tissue [17]. Common phyla identified in the lung of healthy subjects include Proteobacteria, Firmicutes, and Bacteroidetes, while at a genus level, the most commonly found are *Streptococcus*, *Prevotella,* and *Veillonella* [17,19]. The composition of a healthy lung microbiome is dynamic and transient because it depends on several factors [17,19]. Firstly, micro-aspiration from the oral cavity and inhalation from the nasopharyngeal system is the main route of microorganism colonization into healthy lungs [19]. Secondly, microbes move into the airways also from inhalation, direct mucosal dispersion, and a gradient defined by high bacterial biomass in the oral cavity down to a low bacterial abundance in the lung [20]. Thirdly, cilia and innate immune cells are abundant in the respiratory system impacting the speed at which microbes are removed. Indeed, the pulmonary epithelium is composed of ciliated and secretory cells. However, it is not continuous from the upper respiratory tract (UTR) to the alveoli. In the large bronchi, the mucous and serous cells are located in a submucosal gland, whereas moving toward the bronchiole, mucus is produced by club and goblet cells. Type I and II pneumocytes constitute the alveolar epithelium, which secretes a surfactant rather than mucus. In a healthy individual, the mucus layer provides an effective defense against epithelial injury. Thus, altered mucus production and loss of epithelial integrity contribute to several respiratory diseases. Fourthly, airway microbiota composition correlates with age. Aging (>60 years old) is associated with increased Firmicutes and decreased Proteobacteria [21]. Finally, the local environmental conditions, including oxygen partial pressure, temperature and pH fluctuations, nutrient availability are key determinants in lung microbiome composition [17,19]. The pH gradually increases along the respiratory tract from 6.6 in nasal mucosa to 7.1 in the alveoli, whereas the partial pressures of oxygen and carbon dioxide have opposing gradients that are determined by environmental air conditions and gas exchange at the surface of the lungs. These physiological parameters determine the niche-specific selective growth conditions that ultimately shape the microbial communities along the respiratory tract. A shift in the balance of any of these factors can result in an altered microbiome and pulmonary disease [22].

### Challenges in Lung Microbiome Sampling and Analysis

A sampling of the lung microbiome should address key procedural and analytical challenges related to potential contamination from the URT when using bronchoalveolar lavage (BAL) or sputum samples [23]. Moreover, considerable topographic heterogeneity has been observed in the bacterial communities of lung patients with end-stage diseases [24,25]. Regional variation in mucus or surfactant secretion, pH, nutrients, or oxygen availability (e.g., gas trapping) can also increase the variability in the healthy lung microbiome [26]. Moreover, analysis of mycobiome or virome is rarely performed in lung microbiome studies, and both could have a significant impact on the bacterial communities [27,28]. The microbiome might also display a marked spatial variation between sites in the lungs due to genetic or acquired factors [29,30]. In this regard, recent data support a link between air pollution and changes in lung microbiome abundance and diversity [31]. Finally, standardization of methodology is currently limited in respiratory microbiome studies. Developing guidelines on best practices is a current priority to optimize data quality and comparability, similar to that of the International Human Microbiome Standards Project Standard Operating Procedure (SOP)s for stool collection [32].

## 3. The Role of Microbiome in IPF

IPF, the most common idiopathic fibrotic ILD, is associated with a mean survival of 4 years from diagnosis, if untreated [33]. Despite the availability of two anti-fibrotic agents, the prognosis of IPF patients remains dismal, mainly because of the limited knowledge of disease pathogenesis [34]. As its name suggests, the etiology of IPF is unknown. Current disease paradigm is centered on dysregulated wound-healing mechanisms following repetitive and/or persistent alveolar micro-injuries to the alveolar epithelium by environmental triggers (e.g., smoking) in genetically susceptible individuals, leading to fibrosis rather than normal repair [2]. Additional biological mechanisms have been also implicated in IPF pathogenesis, including apoptosis, intra-alveolar coagulation, telomere shortening, and oxidative stress [35,36,37]. In this context, alterations in lung microbiome composition might play key roles in disease pathogenesis and progression. However, it is unknown if alterations in the lung microbiome represent the cause or the consequence of the disease. Indeed, the altered microbiome can be involved in IPF pathogenesis at different steps. Infectious agents can induce alveolar damage, apoptosis, and modulate the host response to injury thus representing the primum movens of IPF development [38]. Some genetic variants commonly associated with IPF, such as the mutant T allele of the MUC5B rs35705950 polymorphism, can facilitate infections and dysbiosis through alterations in the innate immune defense and perpetuate the wound-healing mechanism [5,39]. While many have hypothesized that the anatomic alterations related to the development of fibrosis can facilitate the harboring of the respiratory system by selected microorganisms contributing to the progression of fibrosis, there is no evidence that the extent of fibrosis is related to the bacterial communities [38]. Finally, data from randomized control trial (RCT) showed that immunosuppressive treatments are associated with greater mortality, compared with placebo in IPF patients, suggesting that the modulation of lung microbiome could be of paramount importance in disease progression [40].

### 3.1. The Role of Lung Microbiome during the Natural History of IPF

The first ancillary study that analyzed lung microbiota almost 10 years ago employed the 16S rRNA gene sequencing in a heterogeneous group of ILDs patients, including five with IPF [41]. Although monocentric and based on a small number of samples, this was the first study to demonstrate the presence of bacterial DNA in the lower airways of ILD patients. However, no significant differences in the microbiome between patients with ILD and healthy controls were found. Since then, several studies aiming at further characterizing the lung microbiome in IPF patients have been performed (Table 2).

In the COMET study [42], 55 BAL samples from retrospectively identified IPF patients were evaluated to define the potential contribution of lung microbiota to disease progression. The most commonly identified bacteria were *Prevotella*, *Veillonella,* and *Escherichia* spp. The presence of *Streptococcus* spp. or *Staphylococcus* spp. at baseline was strongly associated with disease progression, defined by a composite outcome including death, acute exacerbation, lung transplant, or relative decline in forced vital capacity (FVC) >10% or diffusing capacity of the lung for carbon monoxide (DLCO) >15%. However, these microorganisms were found in less than half of the study population and, despite their association with a faster progression, a causal relationship with the development of the disease was not established [42]. Furthermore, the absence of a control group limited the interpretation of the results.

A prospective study carried out by Molyneaux et al. aimed at elucidating the role of lung microbiota in the pathogenesis and progression of IPF [43]. This study included 65 patients with IPF, 27 healthy controls, and 17 patients with COPD. The authors found a twofold increase in bacterial burden in lung microbiota (measured as copy number of the 16S rRNA gene·mL^−1^ of BAL fluid) of IPF patients, compared with healthy controls and COPD patients. Additionally, an increased bacterial load at the time of diagnosis was correlated with a more rapid progression of IPF and a higher risk of mortality (HR 4.59). A reduced microbial diversity was also observed in the IPF cohort with an abundance for *Veillonella*, *Neisseria*, *Streptococcus,* and *Haemophilus* spp. Finally, a higher bacterial load was also associated with the carriage of the MUC5B s35705950 T allele, the strongest genetic factor for the development of IPF [3]. The authors concluded that an increased bacterial burden, and not specific populations of bacteria, was able to predict disease progression and mortality in IPF [43].

A single-center retrospective study conducted by Takahashi et al. confirmed that an impaired diversity of lung microbiota was implicated in the progression of IPF [44]. An abundance of Streptococcaceae, Veillonellaceae, and Prevotellaceae families and a decrease in the phylum Proteobacteria in the lower airways of IPF patients led to a reduced microbiota diversity and correlated with disease progression. However, limitations of the study included the lack of a control group, its retrospective nature as well as the small sample size.

Further studies that explored the correlation between microbiome and lung inflammation and/or fibrosis suggested a direct or synergistic role of the lower airways microbiome in driving alveolar inflammation and fibrogenesis [45,46]. In an effort to move from descriptive and observational studies to functional ones, the investigators of the COMET study integrated microbial data with peripheral blood transcriptional profiles [45,46]. Specifically, in a study of 68 patients with IPF, Huang et al. evaluated the correlation of microbial interaction and host immune response with disease progression, in vitro fibroblast function, and leukocytes phenotypes [45]. The authors demonstrated that the abundance of *Prevotella* and *Staphylococcus* negatively correlated with increased expression of host immune-response-related signaling pathways. These data provided additional evidence that innate immune responses are aberrant in IPF patients and may be modulated by alterations in the microbial community. Moreover, downregulation of the host immune response by inhibition of signaling pathways was associated with worse survival. This was the first experience showing that host–microbiome interactions enhance fibroblast responsiveness and reduce patients’ survival. Further analysis of the host transcriptome demonstrated an apparent host response to the presence of an altered or more abundant microbiome, suggesting that it may act as persistent stimuli for repetitive alveolar injury in IPF [47].

Recently, O’Dwyer et al. employed digital droplet polymerase chain reaction (PCR), a more sensitive measure optimized for low-biomass sample load, to reanalyze patients enrolled in the COMET study who had BAL-derived DNA available for analysis [46]. The authors examined the effect of lung microbiota on local alveolar inflammation and fibrosis. Their results confirmed previous findings and showed that a higher bacterial burden is associated with IPF progression. The increased bacterial burden was associated with significant differences in community composition and loss of community diversity. Notably, lung dysbiosis was associated with a pro-inflammatory and pro-fibrotic signal in the airways and aberrant repair. The authors also employed a murine model of bleomycin-induced fibrosis to unravel the mechanisms underlying these abnormalities. In this animal model, lung dysbiosis increased rapidly during the inflammatory phase and persisted during the fibrotic phase. However, the absence of lung microbiota, studied using germ-free mice, conveys a survival advantage after bleomycin exposure, suggesting that lung dysbiosis precedes the development of fibrosis. This translational study demonstrates that the lung microbiota might play causal roles in pulmonary fibrosis progression and may represent potentially treatable traits for preventing the dysregulated repair of IPF. However, the known limitations of the bleomycin-induced models of human IPF reduce the generalizability of these results.

All the studies mentioned so far evaluated lung microbiota of IPF patients on BAL collected on a single-time point. In an attempt to quantify the burden and communities in the fibrotic interstitium, Kitsios et al. carried out the microbiome in lung explants (MiLEs-IPF) using lung tissue samples [48]. Lower-lobe subpleural tissue was obtained from 40 patients with end-stage IPF undergoing transplantation or post-mortem. In contrast with previously mentioned studies that used BAL as the matrix, the authors found a low bacterial signal in the explanted lung similar to those of negative controls and in contrast with the abundance of pathogens identified in lung explants of cystic fibrosis patients. The authors, therefore, concluded that there was no detectable bacterial DNA in lung tissues of end-stage IPF patients. However, several limitations of the study should be considered: (1) subpleural lung regions with advanced honeycombing are considered as inhospitable for bacterial growth; (2) the sample size may not have been adequate, given that 35% of controls had detectable bacterial DNA; (3) end-stage disease may not be representative of the underlying process; (4) controls were represented by donor lungs that were unsuitable for transplantation; (5) it is possible that microbiome plays a role within the airways of IPF but not in the alveoli [49]. Therefore, larger studies on no end-stage ILDs are preferable.

Recently, Yin et al., for the first time, analyzed the virome in patients with stable IPF by using next-generation RNA sequencing [50]. The authors retrospectively analyzed lung tissue samples from 28 patients with IPF and 20 controls who underwent surgical lung biopsy. A sporadic presence of viral RNA in tissue specimens was detected by real-time quantitative PCR, although there were no significant differences between lungs of IPF patients and controls with regard to the abundance of viral RNA. Further studies are needed to evaluate the lung virome of IPF patients and its interplay with host immunity. In this regard, an interactome approach has been proposed to better evaluate the microbiome of bronchiectasis, showing that integrative microbiomics are able to capture microbial interactions, which cannot be appreciated by studying single microbial groups [51].

### 3.2. Lung Microbiome in IPF Patients during an Acute Exacerbation

The occurrence of an acute exacerbation (AE) complicates the natural history of a sizeable minority of IPF patients, and it is associated with a median survival “following the event” of approximately 4 months [53]. The risk for developing an AE-IPF differs across patient subsets. Recent molecular studies identified several biomarkers that are able to predict AE-IPF and may reflect mechanisms of dysregulation underpinning its pathogenesis [54]. Although these episodes have been historically considered as non-infective, the current definition of AE-IPF underlines that episodes of acute respiratory worsening can be either idiopathic or triggered by different factors, including infection [50]. Furthermore, the histological hallmark of AE-IPF is diffuse alveolar damage, which often is histopathologically indistinguishable from an acute lung injury or infection. Due to these reasons, AE-IPF studies on microbiome using non-culture-dependent techniques are of paramount importance.

Molyneaux et al. investigated changes in BAL microbiota using a cohort of 15 stable patients with IPF and 20 patients experiencing an AE-IPF [55]. The authors showed that patients with AE-IPF had higher bacterial loads (up to four times) in comparison with stable-IPF patients. A significant outgrowth was detected for Proteobacteria, in particular *Campylobacter* spp. and *Stenotrophomonas* spp., while a significant decrease was found for *Veillonella* spp. Based on the significant increase in proteobacteria, which are gastric-associated pathogens, in the BAL fluid during an AE, the authors hypothesized a causative role for aspiration in triggering AE-IPF through continuous alveolar epithelial cell injury.

Evidence on the role of virome in AE-IPF is lacking, although viruses can play a causative role [11]. Recently, sequences of 57 viruses were detected in the nasopharyngeal swab of 18 out of 30 patients with AE-IPF, compared with 13 out of 30 patients with stable disease [56]. Moreover, AE-IPF showed increased levels of several pro-inflammatory cytokines, such as interleukin 6 (IL-6), interferon-gamma (IFNγ), and IL-9, compared with IPF patients with stable disease and controls. HHV and influenza virus A was the most common viruses detected in the AE-IPF group. However, nasopharyngeal microbiota is highly different from lung microbiota, and this is an important limitation of the study.

## 4. The Role of Lung Microbiome in Other Interstitial Lung Diseases

### 4.1. Hypersensitivity Pneumonitis

Hypersensitivity pneumonitis (HP) results from an immune-mediated reaction in genetically predisposed individuals who are exposed to an inhaled antigen [57]. Chronic exposure to the inciting antigen may induce a fibrotic remodeling of the lung parenchyma that might be radiologically and histopathologically indistinguishable from IPF, suggesting that profibrotic pathways may be shared by different forms of progressive fibrosis [58]. However, in the early phases of the disease, immunosuppressive therapy is detrimental in IPF but often beneficial in HP, highlighting the existence of substantial pathogenetic differences between the two diseases [56]. In HP, it has been hypothesized that the microbiome may act in synergy with a dysregulated immune system.

In 2020, Invernizzi et al. evaluated the role of microbiota in patients with HP [52]. Moreover, they used IPF patients as diseased controls to test the hypothesis that observed alterations in the lung microbiome are disease specific and do not simply reflect the presence of fibrosis within the lung. In a monocentric study, the authors prospectively recruited 110 patients with chronic hypersensitivity pneumonitis (CHP) and compared their BAL microbiota (sequenced with PCR amplification of the 16S rRNA genes) with those of 45 IPF patients and 28 control subjects. The study showed significant differences in the microbiota composition of CHP patients, compared with IPF patients; CHP patients exhibited lower BAL bacterial loads, compared with IPF patients. With regard to microbiota composition, patients with IPF showed a greater abundance of Firmicutes and a lower abundance of Proteobacteria, compared with CHP patients. At the genus level, *Staphylococcus* was more abundant in patients with CHP, compared with IPF, although this did not translate to worse clinical outcomes. Notably, no association was found between bacterial burden and survival of CHP subjects. This observation is particularly interesting given the growing evidence that bacterial burden is associated with mortality in IPF [42,43,45,46]. Moreover, this paper supports the hypothesis that IPF pathogenesis is clearly impacted by the microbiome in contrast to CHP and that the increased bacterial burden reported in IPF does not simply reflect the extent of underlying tissue fibrosis. Further multicentric, functional, and longitudinal studies are needed to validate these findings and examine host–microbe interactions in patients with CHP.

### 4.2. Sarcoidosis

Sarcoidosis is a chronic inflammatory disorder triggered by unknown environmental/infectious agents in genetically predisposed hosts [59]. The current theory on immunopathogenesis proposes that exaggerated immune response to unidentified inciting antigens leads to granulomatous inflammation [60,61]. Several studies have hypothesized that lung microbial communities are associated with derangements in the local immune response [20,62]. Thus, the lung microbiome might be implicated in sarcoidosis pathogenesis and progression.

The first study exploring lung microbiota in sarcoidosis was carried out by Zimmermann et al. [63]. The authors enrolled 71 sarcoidosis patients, 15 IPF patients, and 10 healthy controls. 16S rRNA gene sequencing was used to characterize the lung microbiota. The authors found a similar α diversity between sarcoidosis patients (3.0 ± 0.52 standard deviation [SD]) and healthy controls (2.8 ± 0.69 SD). Regarding microbial composition, *Atopobium* spp. and *Fusobacterium* were detected more frequently in sarcoidosis samples. A subsequent study by Clarke et al. tried to characterize further the microbiome of several tissues in sarcoidosis patients by analyzing BAL, lymph node, and spleen [64]. The authors analyzed 93 sarcoidosis patients and 72 controls using metagenomic sequencing and found elevated levels of *Cladosporium* (a ubiquitous and saprobic fungus) in single types of sarcoidosis samples, mostly BAL, but limited concordance across sample types.

In conclusion, whether sarcoidosis pathophysiology drives microbiome changes or dysbiosis drives sarcoidosis progression is still unknown. Further studies are needed, in particular analyzing the interaction among microbiome, host immunity, and genetic predisposition in sarcoidosis.

## 5. Microbiome as a Treatable Trait

### 5.1. Antibiotic Treatment

The hypothesis that dysbiosis influences disease progression and outcomes in ILDs has paved the way toward the use of long-term antibiotics in these patients. Retrospective studies showed benefits on clinical outcomes in IPF patients treated with prophylactic azithromycin or doxycycline [65,66,67]. Moreover, the result of a pilot study showed that 3 months of co-trimoxazole (trimethoprim–sulfamethoxazole) treatment was able to improve quality of life and lung function decline in patients with fibrotic ILDs, compared with placebo [68]. A subsequent RCT designed to assess the safety and efficacy of oral co-trimoxazole for 12 months, in addition to usual treatment in patients with fibrotic idiopathic interstitial pneumonia, showed an improvement in health-related quality of life (QoL) and, in those adhering to the study protocol, a reduction in mortality [69]. However, it could be argued that the reduced mortality in the antibiotic group might be attributed to a reduction in the rate of respiratory infections, given that most patients on “usual treatment” were taking immunosuppressants.

More recently, three RCTs have explored the role of long-term antibiotics on relevant outcomes in patients with IPF [70,71,72] (Table 3). In the light of its anti-inflammatory and antimicrobial properties, a double-blind, randomized, placebo-controlled, cross-over clinical trial explored the effect of azithromycin 500mg 3 times per week in a 12-week intervention period [70]. The primary outcome was the change in cough-related QoL measured by the Leicester Cough Questionnaire (LCQ). Among the 25 study participants, no significant change in LCQ with azithromycin vs. placebo was demonstrated. Similarly, there was no significant difference in change in respiratory polygraphy measuring cough frequency. With regard to adverse effects, diarrhea was more frequent in patients treated with azithromycin than placebo (43% vs. 5%; *p* = 0.03).

The EME-TIPAC was a double-blind, placebo-controlled, parallel, randomized trial conducted to evaluate the efficacy of co-trimoxazole in patients with moderate and severe IPF [71]. The primary outcome was a composite outcome including time to death, lung transplant, or first hospital admission. The trial included 342 patients treated at 39 ILDs centers in the UK from April 2015 to April 2019. Patients were randomly assigned to 960 mg co-trimoxazole twice daily for 12 to 42 months versus placebo. Overall, 83% of patients completed the trial with a mean duration of follow-up of 1 year. The study failed to demonstrate a beneficial effect of co-trimoxazole on the primary outcome. However, the microbiome composition was not evaluated.

The CleanUP-IPF study was a multicenter, open-label, randomized trial that compared the standard of care vs. standard of care *plus* antimicrobial therapy with either co-trimoxazole or doxycycline [72]. The study was designed as a pragmatic study since participants in both treatment arms had limited in-person visits with the enrolling clinical center. Visits were limited to assessments of lung function and other clinical parameters at time points prior to randomization and at months 12, 24, and 36. In total, 513 patients were randomized from August 2017 to January 2020 in 35 sites in the USA. Similar to the EME-TIPAC study, no statistically significant difference was found between patients treated with co-trimoxazole or doxycycline vs. standard of care on the primary end time point to first non-elective respiratory hospitalization or all-cause mortality. In this study, blood samples for DNA sequencing and transcriptomics, and oral and fecal swabs for determination of the microbiome communities were collected before enrolment and after study completion. Analysis of these samples might be helpful to determine whether specific microbiome phenotypes might benefit from treatment with co-trimoxazole. Indeed, the failure of RCTs exploring antibiotic treatment in ILDs might be explained by the heterogeneity of the study population. Identification of microbiome phenotypes is of paramount importance to shape the right antibiotic for the right patient. RCTs targeting dysbiosis through a more personalized approach should be designed.

### 5.2. Non-Antibiotic Treatment

Increasing evidence suggests that an interaction between the altered microbiome and host immunity might lead to the development and progression of ILDs. Moreover, some comorbidities, such as gastroesophageal reflux (GER), may contribute to altering the microbiome composition and burden [23]. Thus, non-antibiotic treatment addressing inflammation or GER might result in restoration of microbiome composition and improved clinical outcomes. In a pilot, prospective, cohort study, Wang et al. assessed the efficacy of inhaled interferon-gamma (IFN-γ) as a single therapy in IPF patients [73]. Microbiome composition was analyzed in BAL samples at baseline and after 6 months of treatment. The authors showed that the diversity of the microbiome was not impacted by the treatment. Inhaled IFN-γ led to a reduction in pro-inflammatory and pro-fibrotic cytokines, such as IL-13, IL-6, IL-5, and PDGF-AA. Thus, these data suggest that inhaled IFN-γ can alter aberrant immunological and tissue repair pathways in the lower airway mucosa.

Retrospective data have shown a modest benefit of medical and/or surgical treatment of GER in ILD patients, although this has not been a consistent finding [74,75,76]. Evidence from well-designed RCTs studies is needed to determine the impact of GER-directed therapies on the lung microbiome and clinical outcomes.

## 6. Gut–Lung Microbiome Axis: An Emerging and Intriguing Concept

Although the gut and lungs are anatomically distinct, they are formed from the same embryonic tissue and their mucosal tissues bear commonalities in embryology, structure, and physiology [77]. Potential anatomic communications and complex pathways involving their respective microbiota have reinforced the existence of a gut–lung axis [78,79]. The two-way communication hub between the gut and lungs influences the immune status of both organs. Indeed, there is a clear cross-talk in the gut–lung axis that is vital for maintaining homeostasis and shaping the host immune system [79]. This strong correlation between gut and lung could be also responsible for perpetuating inflammatory damage and for establishing a vicious circle [11,80]. Environmental factors, such as diet and antibiotic treatment can shift the gut microbiota toward the outgrowth of pathogenic bacterial species at the expense of beneficial ones [81]. The generated dysbiosis disrupts tissue and immune homeostasis and is associated with diverse inflammatory diseases, including pulmonary ones [82]. Cross-talk occurs through chemical messengers that are produced directly by microorganisms and by the immune system responses that they trigger [79]. For example, short-chain fatty acids (SCFA)s, which are produced in large amounts by some commensal bacteria, can act as signaling molecules between tissues [77,79]. However, the metabolic profiling of the microbial community of the lungs is incomplete, and the roles of SCFAs as organizers of endogenous lung microbial communities, local actors in the respiratory epithelium and immunity, and systemic mediators remain unclear. Although most lines of evidence indicate the primary direction of cross-talk occurs from the gut to the lung, there remains the possibility of communication in the opposite direction. Chronic lung disorders, such as asthma, COPD, and cystic fibrosis exhibit a dysbiotic airway microbiota as well as components of gastrointestinal perturbation [83,84]. Finally, the dysbiotic gut microbiota has also been associated with dysregulated T-cell responses in the lung, altered expression of fibrosis-related genes, and adult-onset lung fibrosis [85,86]. Although the hypothesis that early life intestinal dysbiosis confers susceptibility to late-onset lung fibrosis in humans is intriguing, little is known about the impact of microbial metabolites on the development of pulmonary fibrosis. The mechanisms through which the gut impacts lung health or disease and vice versa are only starting to be elucidated.

## 7. Conclusions

Preliminary evidence suggests that the lung microbiome might influence the natural history of ILDs. The potential mechanisms by which these changes may drive disease pathogenesis and progression are largely unknown. Few longitudinal studies have been performed thus far, and the relationship between microbiome and ILDs is largely associative rather than causative, making translation to clinical applications challenging. Recent evidence showed that also the gut microbiome has a profound influence on lung diseases. Enhancement of the immune response is likely the mechanism by which the gut microbiome is capable of impacting lung homeostasis. However, the absence of an accurate animal model of pulmonary fibrosis hampers our understanding of disease pathogenesis and microbiome interaction. Untangling the host–microbiome interaction in ILD patients is a current research priority. Multicentric, prospective observational studies are needed to characterize the lung microbiome and its interaction with the immune response of the host in order to define pheno-endotypes. Lung dysbiosis is a potentially treatable trait across several ILDs. Although results from RCTs showed that modification of microbiome through antibiotic administration did not improve outcomes in ILDs patients, proper characterization of microbiome and identification of phenotypes might be crucial to modify the trajectory of these dreadful diseases.

## Figures and Tables

**Table 1:** Current microbiome terminology used [6,13,18].

Term	Definition
Microbiome	The community of commensal, symbiotic, and pathogenic microorganisms within a body space or other environment.
Microbiota	The assemblage of living microorganisms present in a defined environment.
Metagenome	The genetic information of the microbiota, obtained from genetic sequencing that is analyzed, organized, and identified through computational tools, using databases of previously known sequences.
Metabolomics	The analytical approaches used to determine the metabolite profile(s) in any given strain or single tissue.
Metatranscriptomics	Analysis of the suite of expressed RNAs (meta-RNAs) by high-throughput sequencing of the corresponding meta-cDNAs.
Metaproteomics	Large-scale characterization of the entire protein complement of environmental or clinical samples at a given point in time.
OTUs	Clusters of similar 16S rRNA gene sequences. Each OTU represents a taxonomic unit of a bacteria family or genus depending on the sequence similarity threshold.
16S rRNA gene	Component of the 30S small subunit of prokaryotic ribosomes. It is used in molecular studies owing to its extremely slow rate of evolution and the presence of both variable and constant regions.
Dysbiosis	An imbalance in the composition of the microbiota of a given niche, related to changes in local conditions.
Abundance	The total number of bacteria individuals in a specific sample.
Evenness	The measure of similarity in relative abundance/frequency distribution of OTUs within a community.
Richness	The number of different species/OTUs in a specific sample.
α-diversity	α-diversity measures the diversity within a sample diversity and is based on the relative abundance of taxa.
β-diversity	β-diversity is the measure for differences between samples from different groups.
Shannon index	The measure of diversity combining richness and evenness.

Abbreviations: OTU: operational taxonomic unit.

**Table 2:** Studies investigating the role of microbiota in development and progression of IPF using non-culture-dependent techniques.

Author and Year	Design of the Study	Sample Size	Microbiome Assessment	Sample Type	Main Findings	Limitations
Han 2014 [42]	Retrospective, multicenter, observational	55 IPF patients	PCR amplification of the 16S rRNA genes	BAL from right middle lobe or lingular segmental	The most commonly identified bacteria were *Prevotella, Veillonella,* and *Escherichia* spp. The presence of *Streptococcus* spp. or *Staphylococcus* spp. was strongly associated with disease progression.	Absence of a control group. Microbiome analysis was not prespecified. No correlation with inflammation markers. Disease progression is defined by a composite outcome.
Molyneaux 2014 [43]	Prospective, monocenter, observational	65 IPF patients, 17 COPD patients, 27 healthy controls	PCR amplification of the 16S rRNA genes	BAL from right middle lobe	Patients with IPF have a two-fold higher bacterial load in BAL compared to controls and significant differences in the composition and diversity of their microbiota. An increased bacterial load at the time of diagnosis identified patients with more rapidly progressive IPF.	Monocenter. Unexplored correlation between microbiome and inflammation markers.
Huang 2017 [45]	Prospective, multicenter, observational	68 IPF patients	PCR amplification of the 16S rRNA genes	BAL from right middle lobe	The abundance of *Prevotella* and *Staphylococcus* was negatively correlated with increased expression of host immune response-related signaling pathways. Host-microbiome interactions have been shown to enhance fibroblast responsiveness and reduce progression-free survival.	Findings are only associative and cannot prove causality given the study design.
Takahashi 2018 [44]	Retrospective, monocenter	34 IPF patients	PCR amplification of the 16S rRNA genes	BAL from right middle lobe or linguar segment	Loss of diversity of the lung microbiota correlated with IPF progression.	Absence of healthy control. Monocenter and retrospective. Small sample size.
Kitsios 2018 [48]	Case–control	40 end-stage IPF and 37 control	PCR amplification of the 16S rRNA genes	Subpleural lower lobe with advanced honeycombing tissue samples	Low bacterial signal in end-stage lung that was similar to negative control samples.	Single sample. Sample from subpleural tissue with extensive honeycombing. Unexplored correlation between microbiome and inflammation markers.
O’Dwyer 2019 [46]	Prospective, multicenter, observational	68 IPF patients	Droplet digital PCR (ddPCR) for the 16S rRNA gene	BAL from right middle lobe	Higher bacterial burden was associated with disease progression. Alterations in lung microbiome burden, composition, and diversity were associated with derangements in alveolar immunity.	Disease progression defined by a composite outcome (death, acute exacerbation, lung transplant, or relative decline in FVC>10% or DLCO>15%). Absence of a control group.
Invernizzi 2021 [52]	Prospective, monocenter, observational	45 IPF patients, 110 CHP patients, 28 controls	PCR amplification of the 16S rRNA genes	BAL according to SOP	At the phylum level, the prevailing microbiota of IPF was *Firmicutes.* There was association between bacterial burden and survival in IPF.	Monocenter. Considerable differences in patient cohorts. Unexplored correlation between microbiome and inflammation markers.
Yin 2021 [50]	Case–control, multicentric	28 IPF patients, 20 controls	Real-time quantitative polymerase chain reaction (qPCR)	Surgical lung biopsy	Sporadic presence of viral RNA in tissue specimens. No significant differences between IPF and control lung regarding the abundance of viral RNA.	Small sample size. Unexplored correlation between microbiome and inflammation markers.

Abbreviations: IPF: idiopathic pulmonary fibrosis; PCR: polymerase chain reaction; BAL: bronchoalveolar lavage; OTU: operational taxonomic unit; FVC: forced vital capacity; DLCO: diffusing lung capacity for carbon monoxide; COPD: chronic obstructive pulmonary disease; CHP: chronic hypersensitivity pneumonitis; SOP: standard operating procedures.

**Table 3:** RCTs evaluating antimicrobial in idiopathic pulmonary fibrosis.

Study	Design	Sample Size	Intervention	Comparator	Duration	Primary Outcome	Results	Safety
Guler 2021 [70]	Double-blind randomized controlled cross-over trial 1:1	25 patients	Azithromycin 500 mg 3 times per week	Placebo	12 weeks	Change in cough-related quality of life measured by the LCQ	No significant change in LCQ with azithromycin or placebo	Gastrointestinal adverse effects were more frequent with azithromycin than with placebo (diarrhea 43% vs. 5%, *p* = 0.03)
Wilson 2020 [71]	Double-blind, placebo-controlled, parallel randomized trial 1:1	342 patients	960 mg of oral co-trimoxazole twice daily	Placebo	Between 12 and 42 months	Composite outcome including time to death, lung transplant, or first non-elective hospital admission	There were no statistically significant differences in primary outcome and other secondary outcomes including lung function, or patient-reported outcomes	Similar rate of adverse events (mostly gastrointestinal) in co-trimoxazole and placebo group
Martinez 2021 [72]	Pragmatic, randomized, unblinded clinical trial 1:1	513 patients	Co-trimoxazole 960 mg twice daily or doxycycline 100 mg once daily if body weight < 50 kg or 100 mg twice daily if ≥50 kg	No antibiotic (unblinded)	Between 12 and 36 months	Time to first nonelective respiratory hospitalization or all-cause mortality	No significant difference between groups. Moreover, there was no statistically significant interaction between the effect of the prespecified antimicrobial agent (co-trimoxazole vs. doxycycline) on the primary end point	Serious adverse events occurring at 5% among those treated with antimicrobials vs. usual care alone. Adverse events included respiratory events (16.5% vs. 10.0%) and infections (2.8% vs. 6.6%), diarrhea (10.2% vs. 3.1%) and rash (6.7% vs. 0%)

Abbreviations: LCQ: Leicester Cough Questionnaire.
